# The association of radiologic right heart strain indices with the severity of pulmonary parenchymal involvement and prognosis in patients with COVID-19

**DOI:** 10.34172/jcvtr.33094

**Published:** 2024-09-20

**Authors:** Parsa Rouzrokh, Malihe Rezaee, Zahra Mohammadipour, Sasan Tavana, Isa Khaheshi, Ali Sheikhy, Taraneh Faghihi Langroudi

**Affiliations:** ^1^Shahid Beheshti University of Medical Sciences, School of Medicine, Tehran, Iran; ^2^Tehran Heart Center, Cardiovascular Diseases Research Institute, Tehran University of Medical Sciences, Tehran, Iran; ^3^Department of Pulmonary Medicine, Clinical Research and Development Center, Shahid Modarres Hospital, Shahid Beheshti University of Medical Sciences, Tehran, Iran; ^4^Cardiovascular Research Center, Shahid Modarres Hospital, Shahid Beheshti University of Medical Sciences, Tehran, Iran; ^5^Radiology Department, Shahid Modarres Hospital, Shahid Beheshti University of Medical Sciences, Tehran, Iran

**Keywords:** COVID-19, Right heart strain, Pulmonary artery dilation, Right ventricle dilation, Adverse outcomes, Lung involvement severity

## Abstract

**Introduction:**

It has been demonstrated that an increase in the diameter of the right ventricle or pulmonary artery in COVID-19 patients could be associated with the severity of lung involvement and may lead to unfavorable outcomes, particularly in the presence of pulmonary vascular diseases. This study investigated the relationship between these right heart strain features, the extent of lung involvement, and their prognostic values in patients without vascular comorbidities.

**Methods:**

This study selected 154 consecutive patients with positive chest computed tomography (CT) findings and no evidence of concurrent pulmonary disease. Clinical characteristics and adverse outcomes in in-hospital settings were collected retrospectively. Diameters of cardiac ventricles and arteries, along with lung opacification scores, were obtained using CT pulmonary angiography (CTPA) findings, and the association of these variables was evaluated.

**Results:**

An increase in pulmonary artery (PA) to ascending aorta (AO) diameter ratio and lung parenchymal damage were significantly and positively correlated (*P*=0.017), but increased right ventricle (RV) to left ventricle (LV) diameter ratio showed no association with the extent of chest opacification (*P*=0.098). Evaluating the prognostic ability of both ratios using logistic regression and receiver operating characteristic (ROC) analysis proved no significant class separation in regards to predicting adverse outcomes (PA/AO: OR:1.081, *P* Value:0.638, RV/LV: OR:1.098, *P* Value:0.344).

**Conclusion:**

In COVID-19 patients without vascular comorbidities, a higher PA/AO diameter ratio was significantly associated with increased lung involvement severity on CT imaging but not with adverse in-hospital outcomes. Conversely, an increased RV/LV ratio on CTPA did not correlate significantly with adverse outcomes or the severity of parenchymal lung damage.

## Introduction

 Causing over 500 million documented cases and 6 million deaths as of August 2022, the most critical global health issue in the 21st century still lingers on.^1^ While pulmonary complications remain a significant concern regarding the coronavirus disease of 2019 (COVID-19), cardiovascular complications could also be responsible for the exacerbation of the clinical features and outcomes of COVID-19. In this respect, acute myocardial infarction, heart failure, arrhythmia, pericarditis, myocarditis, and thromboembolic events have been reported by various studies.^2^ Right heart strain is one of these outcomes, which often arises due to pulmonary arterial hypertension, and its contributing factors like pulmonary emboli. Dilation of the right ventricle and pulmonary trunk are two of the main computed tomography (CT) features of right heart strain.^3-6^ In accordance with available evidence, both echocardiographic and CT findings have corroborated that right ventricular dilation and hence increased right ventricle (RV) to left ventricle (LV) diameter ratio may lead to worse outcomes in acute respiratory distress syndrome (ARDS), interstitial lung disease (ILD) and pulmonary thromboembolism (PTE).^7-9^ Numerous research investigations have consistently highlighted a potential correlation between the diameter ratio of the pulmonary artery (PA) to the ascending aorta (AO) and unfavorable prognostic outcomes in pulmonary conditions, including but not limited to idiopathic pulmonary fibrosis (IPF) and chronic obstructive pulmonary disease (COPD).^10,11^ As a further matter, it has been indicated in a limited number of studies that the ratios mentioned above may be heavily associated with unfavorable outcomes in COVID-19, particularly mortality rates.^12,13^ The main objective of the present study is to evaluate the association between the described right heart strain CT evidence and the extent of lung involvement in COVID-19 patients without vascular comorbidities. Additionally, the study aims to determine if these findings can predict the likelihood of adverse outcomes for patients with this infectious disease.

## Materials and Methods

###  Study population

 Between July 25, 2020, and October 23, 2021, 175 consecutive patients with positive COVID-19 pneumonia chest CT pulmonary angiography (CTPA) findings obtained in fewer than 35 days from the beginning of clinical signs and symptoms were selected primarily. A total of 21 subjects with evidence of concurrent pulmonary embolism in CTPA or previous history of pulmonary embolism, pulmonary hypertension, and chronic obstructive pulmonary disease were excluded; eventually, 154 patients with negative CTPA for PTE entered the study. This study received approval from the Ethics Committee of the Shahid Beheshti University of Medical Sciences, Tehran, Iran (code number: IR.SBMU.MSP.REC.1399.740). Before enrollment, explicit written consent was obtained from all the participants to participate in the study. All methods and procedures adhered to the applicable guidelines.

###  Data Collection

 Comprehensive data about patient demographics, clinical characteristics, echocardiographic findings, and in-hospital adverse outcomes were retrospectively obtained from medical records. Adverse outcomes were specified as intensive care unit (ICU) admission, intubation, or mortality. The CT scan images were sourced from the picture archiving and communication system (PACS).

###  Chamber quantification on CT images 

**Figure 1:**
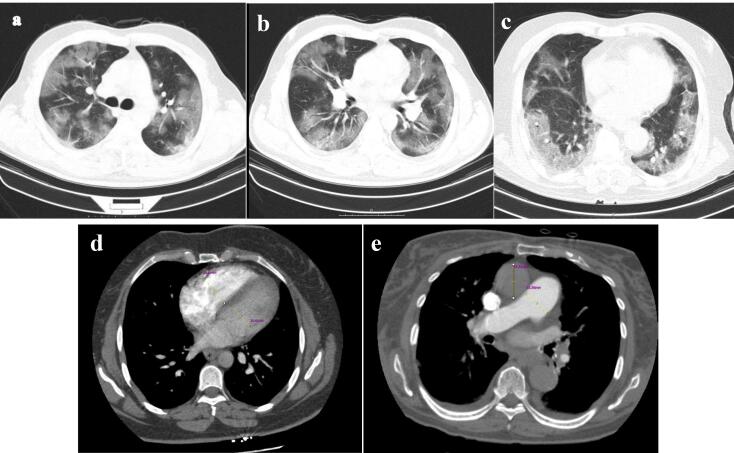
Aquantitative assessment of pulmonary parenchymal involvement was conducted based on lung window images derived from pulmonary CTA. The scoring for parenchymal involvement in this patient is detailed as follows: Right Upper Lobe (RUL): 11, Right Middle Lobe (RML): 4, Right Lower Lobe (RLL): 14, Left Upper Lobe (LUL): 8, Lingula: 6, Left Lower Lobe (LLL): 14, resulting in a total score of 54 out of 190 (28.42%) (figures a, b, and c). Additionally, the computed tomography angiography (CTA) provided quantitative measurements for the right ventricle (RV) with a diameter of 38mm, left ventricle (LV) with a diameter of 30mm, ascending aorta (AO) with a diameter of 34mm, and pulmonary artery (PA) with a diameter of 29mm (figures d and e). CTA computerized tomography angiography, RV right ventricle, LV left ventricle, PA pulmonary artery, AO ascending aorta

 Our cases were scanned by a multidetector CT scanner (Brilliance 64, Philips Medical System, Cleveland, OH, USA). Our CT protocol is as follows: spiral pulmonary computed tomography angiography (CTA), head first supine, caudocranial scan, scan length: 450mm, tube voltage: 120kV, tube current: 350-450mA (adjusted based on the size of patient), slice thickness: 1mm. The pulmonary CTA was performed with an IV injection of 100cc contrast agent (Iodixanol 320 mg/ml). Using the radiology workstation (extended brilliance workspace, Philips Medical Systems Nederland B.V.), the maximum transverse diameter of the right and left ventricles measured just below the level of mitral and tricuspid valves, respectively, in a 4-chamber view, as well as the maximum transverse diameters of the pulmonary artery and the ascending aorta in the axial plane, were retrospectively examined by an expert cardiothoracic radiologist to calculate the exact ratios mentioned in the study (Figure 1).

###  Chest CT severity score

 The modified CT scoring system introduced by Yang et al evaluated the percentage of parenchymal involvement in all lung regions (Figure 1).^14^ To determine the involvement score of each segment, the following criteria apply: 0% is designated as score 0, 1-5% is given score 1, 6-25% is assigned score 2, 26-50% receives score 3, 51-75% is associated with score 4, and 76-100% is attributed a score of 5. Coefficient 1 was used for ground glass infiltration, and Coefficient 2 for consolidation. These mentioned coefficients were multiplied by the involvement score of each lung segment to determine the severity score of involvement in each lung segment. The total CT severity score (CT-SS) will be the sum of the severity score of involvement in each segment. A radiologist reviewed and reported CT findings blinded to patients’ baseline features and laboratory findings.

###  Statistical Analysis

 Data evaluated in the study is considered to be normally distributed. Mean ± standard deviation (SD) has been employed to present descriptive statistics for continuous variables. Fisher’s exact test examined categorical variables, and the T-test was used to evaluate the associations between continuous and categorical variables. Correlations between the ratios and severity of lung involvement were evaluated using Pearson correlation and linear regression analysis. The discriminatory power of both ratios to predict adverse outcomes was analyzed using the receiver operating characteristic (ROC) curve. All mentioned analyses were conducted using R (version 4.2.2) and SPSS (version 27.0 IBM Corp, NY, USA).

## Results

###  Baseline Features, laboratory findings, and CT findings 

 The baseline clinical attributes and significant laboratory results observed at the admission of 154 patients in the study are shown in Table 1. 51.9% of the patients were female, and the mean age was 53.33 ± 13.16. The most common symptoms at the time of admission were cough (89%), shortness of breath (81.8%), fever (70.1%), sore throat (37%), and chest pain (21.4%). The time interval between symptom-onset and chest CT scan was 10.74 ± 4.91 days. The length of hospital stay of the patients in the study was 9.77 ± 6.50 days. Among the medications used, Anticoagulants (98.1%), Corticosteroids (99.4%), and Remdesivir (83.3%) were the most common. In terms of adverse outcomes (Table 2), fifty patients were admitted to ICU, and 11 of them passed away. Evaluating the association between Baseline and laboratory findings and adverse outcomes has shown that levels of LDH (*P* = 0.06), hospital stay (*P* < 0.001), and using Actemra (*P* = 0.02) are significantly related to the presence of adverse outcomes. Chest CT findings examined in this study are also illustrated in Table 2.

**Table 1:** The baseline clinical features and laboratory findings of COVID-19 patients

**Parameters **	**COVID-19 study cohort (n=154)**
Female, n (%)	80 (51.9%)
Age (years)	53.33 ± 13.16
Symptoms, n (%) - Cough - Shortness of Breath - Fever - Sore throat - Chest Pain	137 (89%) 126 (81.8%) 108 (70.1%) 57 (37%) 33 (21.4%)
Symptom-onset → Chest CTA time (days) Length of hospital stay (days)	10.74 ± 4.91 9.77 ± 6.50
Past Medical History, n (%) - CKD - HTN - DM - IHD	1 (0.6%) 32 (20.8%) 27 (17.5%) 14 (9.1%)
Covid medication, n (%) - Chloroquine - Anticoagulant - Corticosteroid - IFN-β - Actemra - Remdesivir - Favipiravir - Pirfenidone	5 (3.2%) 151 (98.1%) 153 (99.4%) 54 (35.1%) 53 (34.4%) 129 (83.3%) 27 (17.5%) 11 (7.1%)
Laboratory data - CRP (mg/dl – 0 to 3) - ESR (mm/hr) - D-dimer (mcg/mL) - Creatinine (mg/dL) - Absolute lymphocytes (/mcL) - Platelets (K/micro-L) - AST (U/L) - ALT (U/L) - LDH (U/L) - PCT (ng/mL)	+ 1: 41 (26.6%), + 2: 59 (38.3%), + 3: 41 (26.5%) 45.07 ± 32.06 1.67 ± 1.83 1.13 ± 0.19 1007 ± 1180 229 ± 91 61.07 ± 43.52 52.86 ± 41.97 643.68 ± 273.85 0.13 ± 0.40

**Table 2:** Supplemental oxygen, adverse outcomes, and chest CT findings of COVID-19 patients

**Parameters **	**COVID-19 study cohort (n=154)**
Supplemental oxygen, n (%) - Nasal cannula - Oxygen mask - BiPAP	85 (55.2%) 82 (53.2%) 24 (15.6%)
Adverse outcomes, n (%) - ICU admission - Intubation - Death	50 (32.5%) 10 (6.5%) 11 (7.1%)
Chest CT Findings - CT-SS - Lung involvement (%) - PA to A ratio - RV to LV ratio	41.38 ± 24.89 21.77 ± 13.10 0.77 ± 0.12 0.95 ± 0.15

###  PA to AO and lung parenchymal involvement

 As previously mentioned, the Lung parenchymal involvement of patients in this study was measured using the CT scoring system, and the ratios are derived from CTA findings. Evaluating the association between the pulmonary artery to ascending aorta diameter ratio and the severity of lung involvement has shown a statistically significant and positive correlation between these two variables. The linear regression analysis indicates that the participant’s severity score increased by 3.969 for each unit of the mentioned ratio (95% CI: 0.723-7.216, *P* = 0.017).

###  RV to LV and lung involvement

 Contrary to the prior ratio, the right ventricle to left ventricle maximum transverse diameter ratio is not statistically correlated to parenchymal involvement of lung regions. The results of the linear regression analysis showed no significant association and correlation between the dilation of the right ventricle and CT-SS of the patients in the study (p = 0.098, 95% CI: -0.409-4.769).

###  PA to AO and prediction of in-hospital adverse outcomes 

**Figure 2:**
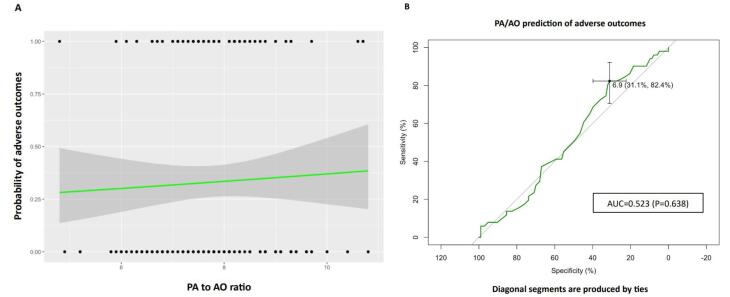
The pulmonary artery to ascending aorta (PA/AO) ratio was analyzed to assess its correlation with adverse outcomes, specifically ICU admission, intubation, or mortality. A logistic regression plot was generated, where dots on the graph represent data points: upper dots indicate the presence of adverse outcomes, and lower dots indicate the absence of adverse outcomes. The X-axis denotes the PA/AO ratio, while the Y-axis illustrates the probability of adverse outcomes. The logistic regression fit is depicted by the black line, and the gray zone represents the 95% confidence interval. Additionally, a Receiver Operator Curve (ROC) was constructed to evaluate the predictive capacity of PA/AO for adverse outcomes. The Area Under the Curve (AUC) was determined as 0.523 (p = 0.638, 95% CI: 0.429-0.618), with a specificity of 31.1% (22.3%-39.8%) and sensitivity of 82.4% (70.6%-92.2%). The optimal cutoff value for PA/AO was identified as 1.1. ICU intensive care unit, PA pulmonary artery, AO ascending aorta

 ICU admission, intubation, and death are the adverse outcomes evaluated in the study (Table 2). Based on logistic regression analysis, there was no significant association between higher ratios of PA/AO and the presence of adverse outcomes (OR: 1.081, 95% CI: 0.187,1.429). Evaluating the predictivity of the PA to AO for at least one of the three outcomes using the receiver operating characteristic (ROC) curve analysis has shown that increased PA could not be considered a robust prognostic factor for adverse outcomes in the patients. As illustrated in Figure 2, ROC of PA/AO predicting adverse outcomes suggests that this model has no class separation capacity [PA/AO ratio: AUC = 0.523, *P* = 0.638, 95% CI: 0.429-0.618, specificity:31.1% (22.3%-39.8%) Sensitivity:82.4% (70.6%-92.2%), with an optimal cutoff of 1.1 for PA/AO].

###  RV to LV and prediction of in-hospital adverse outcomes

**Figure 3:**
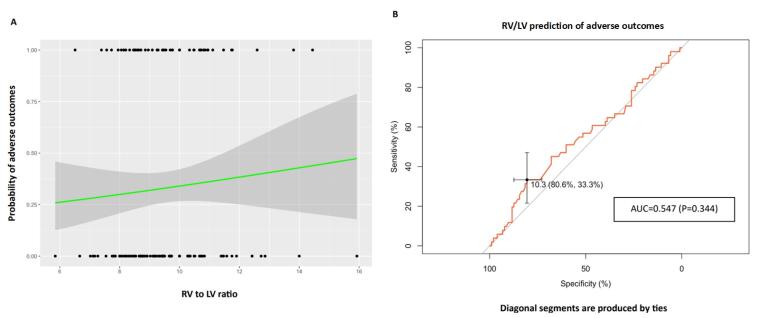
The right ventricle to left ventricle (RV/LV) ratio was evaluated to examine its association with adverse outcomes, encompassing ICU admission, intubation, or mortality. A logistic regression plot was constructed, where data points are represented by dots: upper dots signifying the presence of adverse outcomes, and lower dots denoting the absence of adverse outcomes. The X-axis reflects the RV/LV ratio, while the Y-axis illustrates the probability of adverse outcomes. The logistic regression fit is depicted by the black line, and the 95% confidence interval is represented by the gray zone. Furthermore, a Receiver Operator Curve (ROC) was generated to assess the predictive performance of RV/LV for adverse outcomes. The Area Under the Curve (AUC) was determined as 0.547 (p = 0.344, 95% CI: 0.448-0.646), with a specificity of 80.6% (72.8%-88.4%) and sensitivity of 33.3% (21.6%-47.1%). The optimal cutoff value for RV/LV was identified as 1.1. RV: right ventricle, LV: left ventricle, ICU: intensive care unit

 Like PA to AO, the right ventricle to left ventricle diameter ratio is not correlated with at least one of the adverse outcomes (OR: 1.098, 95% CI: 0.883,1.366). Evaluating the prognostic value of this ratio using ROC analysis has also shown no significant class separation, as illustrated in Figure 3 [AUC = 0.547, *P* = 0.344, 95% CI: 0.448-0.646, specificity:80.6% (72.8%-88.4%), Sensitivity:33.3% (21.6%-47.1%) with an optimal cutoff of 1.1 for RV/LV].

## Discussion

 Our findings in the ongoing study indicate that the PA/AO diameter ratio was positively related to the severity of lung involvement on CT imaging in COVID-19 patients, with the severity score increased by 3.969 for each unit of the PA/AO diameter ratio. In contrast, a notable correlation was absent between the RV/LV maximum transverse diameter ratio and the severity of lung involvement. Further analyses have revealed no significant association between the mentioned ratios and unfavorable outcomes, including ICU admission, intubation, and in-hospital mortality, in COVID-19 patients without pulmonary vascular comorbidities.

 The cardiac involvement and manifestations in patients with COVID-19 have been established, including heart failure, cardiogenic shock, myocardial infarction, arrhythmia, and myocarditis, which are associated with poor prognosis.^15,16^ Numerous studies demonstrated that acute myocardial injury could occur in about 20-30% of hospitalized COVID-19 patients.^17,18^ Several mechanisms proposed to play a role in the pathogenesis of COVID-19 infection-induced myocardial injury, including direct cardiomyocyte damage, systemic inflammation, cytokine storm, hypoxia, and induction cardiac fibrosis-related processes.^19,20^ RV involvement, such as RV dilation, has been observed frequently in admitted COVID-19 patients. Also, based on the available evidence, RV involvement is more prevalent than LV involvement among these patients.^21^

 Furthermore, it has been demonstrated that COVID-19 infection induces a hypercoagulability state, mainly due to stimulating the inflammatory processes, which makes patients more susceptible to venues and arterial thrombotic events, such as PTE.^22,23^ A meta-analysis study indicated that the occurrence rate of PTE among COVID-19 patients was 16.5% (95% CI: 11.6, 22.9) with a significantly higher frequency observed in patients admitted to the ICU compared to those who were not (24.7% vs. 10.5%).^24^ Moreover, pulmonary hypertension is reported among patients with COVID-19, which could be related to the presence of PTE, thrombotic microangiopathy, lung parenchymal damage, and hypoxia. COVID-19 patients who have pulmonary hypertension were observed to have a higher risk of poor clinical prognosis.^25^

 The growing body of studies has proven that PTE can cause RV enlargement and increased RV/LV diameter ratio, particularly in severe cases.^26^ In this way, the results of Cho et al’s study revealed that the RV/LV > 1 diameter ratio was significantly associated with a higher ICU admission rate, compared with RV/LV < 1 (28.05% vs. 11.61%) in patients with acute PTE. They concluded that the RV/LV diameter ratio, measured based on CTPA, could be a prognostic factor for the severity of PE.^27^ Similarly, Spruijt et al showed that RV/LV ratio ≥ 1.2 had a predictive value for unfavorable outcomes in patients with pulmonary hypertension.^28^

 Tao et al evaluated the RV/LV ratio and its prognostic value among patients with COVID-19. Their findings revealed a significant elevation in the CTPA-based RV/LV in COVID-19 patients compared to the control group. Also, in contrast to our results, they found a notable positive correlation between RV/LV ratio and adverse outcomes, with 45% and 20% of patients with RV/LV ratio ≥ 1.1 and < 1.1, 9/20 experiencing adverse outcomes, respectively. Moreover, RV/LV ratio ≥ 1.1 [AUC = 0.707 (CI: 0.592–0.823, *P* = 0.002)] was suggested as a predictor of adverse outcomes. Despite adverse outcomes, the increased RV/LV ratio was out of proportion to the severity of lung parenchymal injury based on CT scoring, which aligns with our results.^12^

 In coherence with our results, Eslami et al reported that increased PA/AO was positively associated with extensive lung involvement (*P* = 0.001). At the same time, PA/AO > 1 showed an insignificant rise in odds of mortality (OR = 1.9 and *P* = 0.36) in COVID-19 patients.^29^ Conversely, another investigation on COVID-19 affected patients revealed that despite the elevated PA diameter at admission being correlated with mortality, increased PA/AO had no relationship with adverse outcomes.^13^ Regarding the underlying explanation, PA/AO ≥ 0.92 was found to be associated with parenchymal lung damage,^30^ which is the primary characterization of COVID-19 infection, resulting from excess inflammatory responses, microvascular thrombosis, and alveolar damage.^31,32^

 Hayama et al demonstrated that COVID-19 patients who experienced severe respiratory exacerbation had a higher PA/AO diameter ratio (0.97 ± 0.11 vs. 0.82 ± 0.10, *P* < 0.001). A PA/AO ratio greater than 0.9 was notably linked to a heightened risk of respiratory failure and in-hospital death compared to those with a PA/AO ratio of ≤ 0.9 (*P* < 0.001).^33^ Previous studies demonstrated that the PA/AO ratio > 0.9 and 1.0 is considered a marker of pulmonary hypertension and also displayed an increased risk of poorer prognosis in patients with pulmonary hypertension and PTE.^34,35^

 It should be noted that in previous studies which evaluated the RV enlargement, RV/LV, PA/AO, and their prognostic values, the difference between these results between patients who had and did not have PTE has not been determined. In this study, we found no significant correlation between RV/LV and PA/AO ratio and adverse outcomes, in COVID-19 patients without PTE in CTPA and/or history of previous pulmonary hypertension. Therefore, it could be concluded that the relationship between RV/LV and PA/AO ratio with unfavorable prognosis in COVID-19 might have resulted from the presence of concurrent PTE and/or previous history of pulmonary hypertension. As for the severity, the PA/AO ratio showed a significant correlation with the extent of lung involvement, suggesting that pulmonary arterial changes are more directly related to the severity of lung disease in COVID-19, most likely due to the alveolar damage and resultant new pulmonary hypertension caused by the disease. However, the RV/LV ratio did not correlate significantly with lung involvement. This indicates that right ventricular dilation might not be as sensitive a marker for lung involvement severity in COVID-19 patients without pre-existing pulmonary vascular conditions. It is also possible that RV dilation requires more prolonged pressure overload than what was observed in our study.

 On the other hand, Dam et al showed that COVID-19 patients exhibited a lower mean RV/LV ratio based on CTPA (mean difference −0.23, 95% CI (−0.39 to −0.07)) and a decreased prevalence of an RV/LV ratio > 1.0 (26% vs. 49%) compared to control participants.^36^ Therefore, it seems that limited and contradictory data are available regarding the RV/LV ratio and its prognostic value in COVID-19 patients with or without PE. In this line, we suggested that further studies are needed to determine the exact relationship between the RV/LV ratio and outcomes in COVID-19 patients.

 Our study has a number of limitations. Above all, this study is conducted in one center with a small sample size, and the randomized control group was not considered. Calculating SOFA (Sequential Organ Failure Assessment) or APACHE (Acute Physiology and Chronic Health Evaluation) scores was also not feasible due to limitations in the patients’ records. Additionally, echocardiographic parameters such as RV and LV diameters, were not available for most patients. Therefore, despite our findings being susceptible to heartbeat artifacts, and the infeasibility of using cardiac gating due to the unstable heart rates of most of our patients and their high risk of radiation exposure, CTPA was considered the most appropriate procedure. Although the CTPA was conducted during the most severe phase of our patients to rule out pulmonary embolism, the retrospective design of the study prevented us from shortening the timeframe for CTPA acquisition. Since the study aims to investigate the in-hospital adverse outcomes of the patients, no long-term, mid-term, or short-term follow-ups were considered.

## Conclusion

 In this retrospective study, elevated PA/AO diameter ratio was significantly related to the severity of lung involvement on CT imaging in COVID-19 patients without pulmonary vascular diseases, in contrast to adverse outcomes. Also, an increased RV/LV ratio on CTPA in these patients showed no significant correlation with adverse unfavorable outcomes (ICU admission, intubation, or in-hospital death) and severity score of parenchymal lung damage based on chest CT. However, more studies with larger populations and longer follow-ups are needed to determine the prognostic values of these right heart strain findings in patients with COVID-19, considering the possible confounder effects of vascular diseases.

## Acknowledgements

 Not applicable.

## Competing Interests

 The authors declare no conflict of interest.

## Ethical Approval

 This study was approved by the Ethics Committee of the Shahid Beheshti University of Medical Sciences, Tehran, Iran (code number: IR.SBMU.MSP.REC.1399.740). Written Informed consent to participate in the study was obtained from all the participants before enrolment. All methods were performed in accordance with relevant guidelines.
